# Organizational arrangements as a key to enhancing innovativeness and efficiency – analysis of a restructuring hospital in Finland

**DOI:** 10.1186/s12913-022-08376-6

**Published:** 2022-08-10

**Authors:** Anu Kajamaa, Pia Hurmelinna-Laukkanen

**Affiliations:** 1 Faculty of Education, University of Oulu, P.O.Box 8000, 90014 Oulu, Finland; 2 Oulu University School of Business, University of Oulu, P.O.Box 8000, 90014 Oulu, Finland

**Keywords:** Restructuring, Change, Innovativeness, Efficiency, Organizational arrangements, Health care

## Abstract

**Background:**

Challenged to innovate and improve efficiency both at the policy level and in everyday work, many health care organizations are undergoing radical change. However, in many earlier studies, the significance of individuals’ perceptions of their organization and its innovativeness and efficiency during restructuring is not well acknowledged. Our study examines how various organizational arrangements; performance-, hierarchy-, tradition-, and leader-focused types, as well as collaborative and fragmented ones, connect to reaching innovativeness and efficiency in health care during restructuring.

**Method:**

We built on previous organization and management research, innovation studies, and on research focusing in health care restructuring, and conducted an exploratory quantitative case study in a public sector hospital in Finland. Data comprising 447 responses from 19 professional groups across the hospital was analyzed using hierarchical regression analysis.

**Results:**

Our results demonstrate that multiple, co-existing organizational arrangements can promote innovation and efficiency. The perceptions of the organizational members of the nature of their organization need to be generally positive and reflect future-orientation to show positive connections with efficiency and innovativeness; fragmentation in the members’ perceptions of the character of their organization and their inability to go beyond established organizational traditions pose risks of inefficiency and stagnation rather than fruitful exploration. Our study further shows, somewhat surprisingly, that while collaborative organizational arrangements are positively related to increases in perceived efficiency, the same does not apply to innovativeness.

**Conclusions:**

Our study addresses understudied, yet inherently important aspects in providing high-quality health care: the relationships between different organizational arrangements and exploitation and exploration-related outcomes. In particular, examination of individuals’ perceptions (that may have even more weight for the subsequent developments than the actual situation) adds insight to the existing knowledge that has addressed more objective factors. Implications on how to support high levels of performance are drawn for management of professional and pluralistic organizations undergoing restructuring. Our findings also generate information that is useful for policy making concerned with public sector health care.

## Introduction

Contemporary organizations providing health care services find themselves in a challenging situation. Recently, the COVID-19 pandemic has brought unprecedented disruptions and breakdowns, which have generated policy changes and set requirements for the restructuring of health care training programs and care practices [1, 2]. Many health care organizations are required to respond to the contemporary (grand) challenges by transforming and restructuring to be able to exhibit innovation and efficiency simultaneously. Under such pressures, many organizations have made efforts to develop new ways of organizing. However, the pluralistic nature [3, 4] of these contexts, including a complex network of professionals with specific expertise, diverse logics and goals, and complex power relations [5–7] creates challenges, making it difficult to move on with restructuring and other change efforts. Hence, new approaches are needed to understand how innovativeness and efficiency can be promoted under reconstruction.

The previous literature has documented that organizational restructuring causes disruptions and unanticipated consequences, and that finding the best ways to reach the desired outcomes is not always clear to those executing and living through the changes [8–10]. The state of transformation and flux is intricately intertwined with structuring and organizing of working relations, as well as transforming organizational culture and climate, and their management [11, 12]. Despite a proliferation of research on change and restructuring in health care [13–15], so far little research attention has been directed to the role of organizational arrangements within the organizational restructuring processes; the significance and relationships of subjective individual-level perceptions is not fully acknowledged in the health care context.

To understand the relevance of the restructuring efforts for supporting high levels of performance and organizational change, it is important to direct research attention to the different organizational arrangements and how they connect to innovativeness and efficiency in the contemporary health care settings. Our motivation for this study particularly stems from the fact that the literature still lacks comprehensive understanding of the individual-level perceptions that become pivotal when aiming at restructuring that entails both innovativeness and efficiency. In this study, we combined insights from the literature on organizational and management studies [12, 16, 17], innovation research [18, 19], and health care studies focusing on health care restructuring [6, 20]. Adler and Heckscher’s [12, 16] theorization on working relations, built on sociological theories of Weber [11], suggests that working relations emerge in different forms and reflect the logic of associated with the organization, and that they play a role in how well the organization can reach its goals. We have built on these notions, focusing specifically on the individuals’ perceptions and views of organizational practices, processes, culture, climate, values, and relationships as relevant factors; individuals need to be able to function in ways that match their views of their organization and experience that their values, match the values of the organization [3, 12, 21, 22]. In fact, individual-level perceptions – by which we mean the verbally expressed views, understandings, and efforts of sensemaking – may have even more power for the subsequent developments in an organization than the actual situation [3, 22].

Our specific focus is on various *organizational arrangements* [12, 16, 23], that is, the perceptions of the organization’s members on organizational and working practices, processes, climate, values, relationships, and culture, that jointly reflect their views on their organization. So far little research attention has been directed to what these perceptions comprise and how they are connected to perceived efficiency and innovativeness, especially under organizational restructuring. To narrow the gap in the existing research, as our research question we ask: *How can organizational arrangements connect to reaching innovativeness and efficiency in a restructuring organization*? With this research interest in mind, we investigated a public sector hospital located in Finland and undergoing a notable renovation and structural changes, pressuring the staff to improve its performance. With the help of a survey, we explored quantitatively the relationships between different organizational arrangements—which we categorized by following and modifying Adler & Heckscher’s [12, 24], classification of working relations—and efficiency and innovativeness, as perceived by the organization’s members representing diverse interests and professional groups.

### Theoretical background

Contemporary organizations frequently face challenges raised by multiple demands from varying stakeholders, technological developments, and cross-sectoral convergence [25]. There is an increasing need to master the complex management of the *twin requirements of efficiency and innovation* [17, 26] – that is, to competently balance between exploitation (gaining short-term benefit) and exploration (the development of new ideas and opportunities) [27–29]. Accordingly, a notable amount of theorization exists on how to reach the goals of efficiency and innovativeness simultaneously [26]. Existing research has reported organizations’ abilities to exploit existing capabilities and explore new opportunities to occur increasingly in interactive activities with varied actors, both within and across organizations [30–32]. Prior studies have also raised related challenges and suggested that sustaining two different but intertwined organizational logics is a difficult learning challenge that typically requires time and strong commitment [6, 7, 33].

*Organizational restructuring* has been found to be an enabling factor to concurrent innovativeness and efficiency that, in turn, provides organizations with favorable performance outcomes [7, 17–19]. Literature focused on restructuring has, for example, examined its processes and issues such as resistance and diffusion of new practices throughout the organization. However, challenges exist as the actors involved in change efforts may perceive demands for efficiency and innovativeness in quite different ways, making it difficult to move on with change especially in pluralistic organizations, such as health care organizations.

In the search for understanding these issues, research has turned to *working relations and organizational culture* as factors that connect (re)structuring and balancing between efficiency and innovativeness [12]. Most often, these issues are considered at the level of (governance of an) organization (or its divisions) [4]. Complementing studies such as that by Slåtten and colleagues [34], we turn our attention to the *individual level*. Recently, it has been shown that organizations with multiple goals, or even internal disagreement and imbalance, are able to proceed with change [3] *if* the ways in which the organization is arranged allows this. In many cases, the question is about an organization’s members being able to function in ways that match their views of the organization; that is, in ways that accommodate the organization’s members’ perceptions of its central facets like relationships, values, processes, and culture [3, 12, 21, 22].

During change and restructuring, some elements are often held while others are replaced. Adding to the complexity, hospitals are directed by well-defined health care plans, policies, and regulations, which are perceived differently by different actors in these contexts [5, 35]. Therefore, it is likely that *varying perceptions will co-exist* among individuals on the organization and its tenets even more than during more stable times. The effects of these perceptions are not always obvious [8, 12, 15, 22, 36]. Our motivation for this study stems from the fact that the existing literature still lacks a comprehensive understanding of the individual-level perceptions that become pivotal when aiming at restructuring that promotes both innovativeness and efficiency [12, 37].

These issues are particularly prevalent in the health care sector [13] in which organizing and restructuring for different combinations of expertise and work are relevant means to reach beneficial *innovation and efficiency outcomes* [6, 14, 30, 38]. Miller and French [6] (p. 1534) indicate hospitals “occupy a vexed position in the policy landscape [because the hospitals] must orchestrate a range of interests that may not always coexist harmoniously.”

Scholars studying the transformation pressures of health care organizations, such as public sector hospitals [39, 40], have stated, first, that *innovation* is important as it contributes to better clinical performance in healthcare [12, 14, 20, 41, 42]. Currently many hospitals aim to foster learning, creativity, and entrepreneurial attitudes among their employees [5, 15, 43] to promote hospitals’ innovative capability [44] and innovation performance [45].

However, at the same time, strong differentiation and specialization of hospitals has increased the costs of health care provision, thereby starting the search for cost savings through increased *efficiency* [7, 46]. In public sector hospitals, restructuring efforts are often conducted by merging small units into large-scale organizations with the aim of reducing expenditure and labor expenses [14, 47].

Managing the simultaneous efficiency and innovation requirements can be difficult in health care, and the attempts to change and create innovation often fail [6, 48]. There are notable uncertainties related to the radical changes and restructuring processes in the health care sector [6, 26, 49], which complicate the relationships between innovation and efficiency, and organizational arrangements. This raises the question of how organizational arrangements connect to exploitation and exploration-related outcomes.

### Hypothesis development

Looking into the individual organizational arrangements may help understand their connection with innovativeness and efficiency better. Elucidating the forms of working relations, Adler and Hecksher [12, 16] advocate that competitive market or legal-rational bureaucratic forms of these relations are present when organizational actions follow the organizational form of instrumental rationality. A top-down approach to organizational change is dominating, and should enhance efficiency, especially in relatively stable organizational environments. Nevertheless, the market- and bureaucratic logics have some relevant differences. When executed well, what Adler and Hecksher [12] call *market orientation* can be quite beneficial not only for efficiency, but also for innovativeness [50, 51].

We follow this view, and suggest that employees can be motivated and be provided with the structures they need during change by a *performance-focused organizational arrangement* that involves the perception and expectation of the organizational members that goals are set by the top management, and that employees are free to use their creativity regarding the means [12, 37, 52]. In particular, when members of the organization consider their organization to have a performance focus, they perceive its values as reflecting appreciation of good performance, expect rewards to follow from achieving the set goals, and accept a certain level of competition being present in the relational interaction – all of which may promote efficiency and innovativeness [6]. As to limitations, Adler and colleagues [53] suggest that market orientation easily fails in complex organizations in which activities are interdependent, rather than independent, and in which both efficiency and flexibility are needed to achieve organizational change. As these aspects also apply to public sector health care organizations, we suspect that performance-focused organizational arrangements may become challenged in such contexts. However, as performance-focused organizational arrangements generally highlight appreciation of performance with regard to goals, we expect individuals’ perceptions that their organization has this feature to connect positively with their perceptions about innovativeness and efficiency:

- Hypothesis 1a: Performance-focused organizational arrangement and innovativeness are positively related.
- Hypothesis 1b: Performance-focused organizational arrangement and efficiency are positively related.

However, certain levels of bureaucracy may also be a strength [54]. As understood in earlier studies, bureaucracy refers to expectations that the organization’s members “take the organization’s purpose as given, and […] behave in an instrumentally rational way […] to implement […] procedures as efficiently as possible” [12] (pp. 88). Efficiency may be reached if the organization’s members perceive their organization as having such features and act accordingly.

We build on these ideas and suggest that *hierarchy-focused organizational arrangement* connects to how efficiency-related goals are seen to be met. While this is somewhat different from bureaucracy as considered by Adler and Heckscher [12], we suggest that when the members perceive that they can rely on specific protocols and processes, and that their superiors also expect that kind of behavior, they may find that the organization in general works well, and that its efficiency and innovativeness can be secured [52]. The challenge is that bureaucracy embedded in the hierarchy-focused organizational arrangements, especially if widely spread and highly dominating, easily impedes the flexibility needed for innovation to develop [55]. In line with this, we formulated the following hypotheses:

- Hypothesis 2a: Hierarchy-focused organizational arrangement and innovativeness are negatively related.
- Hypothesis 2b: Hierarchy-focused organizational arrangement and efficiency are positively related.

Moving beyond instrumental rationality (and market and bureaucracy forms within), in their studies on health care, Adler and Heckscher [12, 16] found that the *traditionalistic* form of working relations dominates when an organization is guided by customs defined by its past, and when values reflect its members’ reliance on traditionalistic ties (i.e., a *clan* type of an organization prevails).

We consider that in health care, perceiving professional tradition and loyalty, especially among medical doctors [7, 55] as a leading approach, provides an example of *a tradition-focused organizational arrangement*. With an aim to reduce complexity, health care organizations often favor traditional organizational models, ties, and evaluation methods that push for stability and optimization [56]. Embracing autocratic power, the tradition-focused form highlights organization and systems, such as reward systems, to reflect perceptions of status-acknowledgement and loyalty of members as dominating tenets. This may challenge efficiency, as tradition and focus on maintaining stability tend to be appreciated over the search for leaner structures and ways of operation. A tradition-focused form can help hospitals to avoid errors – which obviously is critical in the field, but at the same time, this approach can easily become inadequate in the contemporary innovation-oriented society [57]. Therefore, we hypothesize the following:

- Hypothesis 3a: Tradition-focused organizational arrangement and innovativeness are negatively related.
- Hypothesis 3b: Tradition-focused organizational arrangement and efficiency are negatively related.

Distinct from the above-described arrangements, *leader-focused organizational arrangement* that we suggest is based on transcendent values, collective emotional (rather than rational) bonds, and commitment to organizational purposes and visions set by exemplary leader(s) [58, 59]. In the work of Adler and Heckscher [12, 16], the corresponding *charismatic* (or affectual in Weber’s [11] terms) form of working relations typically emerges in environments with low functional specialization, and a simple hierarchy centered on a *stand-alone* leader trusted by the subordinates. It may be also relevant in smaller entities within pluralistic organizations. Examples of this in health care include the charismatic *star doctors* who exercise individual authority and power over the system [16]. When organizational members express that they perceive leader-focused organizational arrangement, they are likely to have found the means to embrace radical innovation and to work well in organizational change situations, especially in relatively small and well-defined organizations placing the individual leader and his/her personal appeal at the center. In particular, innovativeness can benefit from individuals who actively promote innovation and remove its barriers by restricting opposition of incumbent actors [60]. However, sometimes actions of an influential individual can be problematic and emerge as dominance, which may bias innovative activity [61]. Nevertheless, as the leaders’ good example can have some relevance, we start our exploration with the following hypotheses:

- Hypothesis 4a: Leader-focused organizational arrangement and innovativeness are positively related.
- Hypothesis 4b: Leader-focused organizational arrangement and efficiency are positively related

Earlier research has suggested that organizational restructuring for efficiency and innovation calls for a shift toward increasing collaboration across boundaries [62, 63]), and Adler and Heckscher, [64] propose that the form of working relations that involves crossing boundaries can be labelled a *collaborative community*.

Capturing this insight, the *collaborative organizational arrangement* is grounded in Weber’s [11] value-rational type of social action, but further overcomes its conventional scale limitations, and is thus especially suitable for dynamic, committed, and collegial [65] organizations. In contexts such as hospitals, with separate work units and multiple professional groups, this organizational arrangement refers to open dialogue and common orientation to shared values (e.g., restoring the health of the patient), mission, and reward systems. The collaborative organizational arrangement – like the collaborative community – indicates that the organization’s members perceive consensus, equality, and commitment to shared views and common values to dominate [22, 66, 67]. A shared purpose and institutionalized dialogue can help achieving efficiency and innovativeness within organizations [6] and building bridges between the top-down and bottom-up approaches to organizational change [12, 30, 36, 64, 68]. The following hypotheses to explore capture this logic:

- Hypothesis 5a: Collaborative organizational arrangement and innovativeness are positively related.
- Hypothesis 5b: Collaborative organizational arrangement and efficiency are positively related.

However, collaboration is not always easy because of the high level of complexity, professional boundaries, hierarchies, and pluralism characteristic in health care [37, 62]. As Adler [23] (pp. 466) states, a collaborative community can rarely be seen “in pure form, and where we do see it, it is typically fragmentary and corrupted by valorization pressures, and always precarious.” Thus, a risk is involved in organizational restructuring, that pursuing collaborative forms will fail and lead to opposite results, such as increased fragmentation [69, 70].

*Fragmentation* can be defined as the perception of lack of the organization’s ability to define a common purpose or preserve its salience [16]. This is not always a completely detrimental issue. It has been stated in previous research that some divergence, dissonance, and certain amount of chaos during organizational restructuring can also generate genuinely new ideas [36, 71]. Thus, fragmentation does not automatically become a barrier to change [3]. However, if the perception of employees is that their organization is fragmented, there is a risk that the common goals will not be reached [72]. We suspect that in most cases, fragmentation is harmful for efficiency and innovation, especially if fragmentation resulting from failing to reach adequate levels of shared purpose emerges in the organizational restructuring processes. Based on these considerations, we present the following tentative hypotheses:

- Hypothesis 6a: Fragmented organizational arrangement and innovativeness are negatively related.
- Hypothesis 6b: Fragmented organizational arrangement and efficiency are negatively related.

**Fig. 1:**
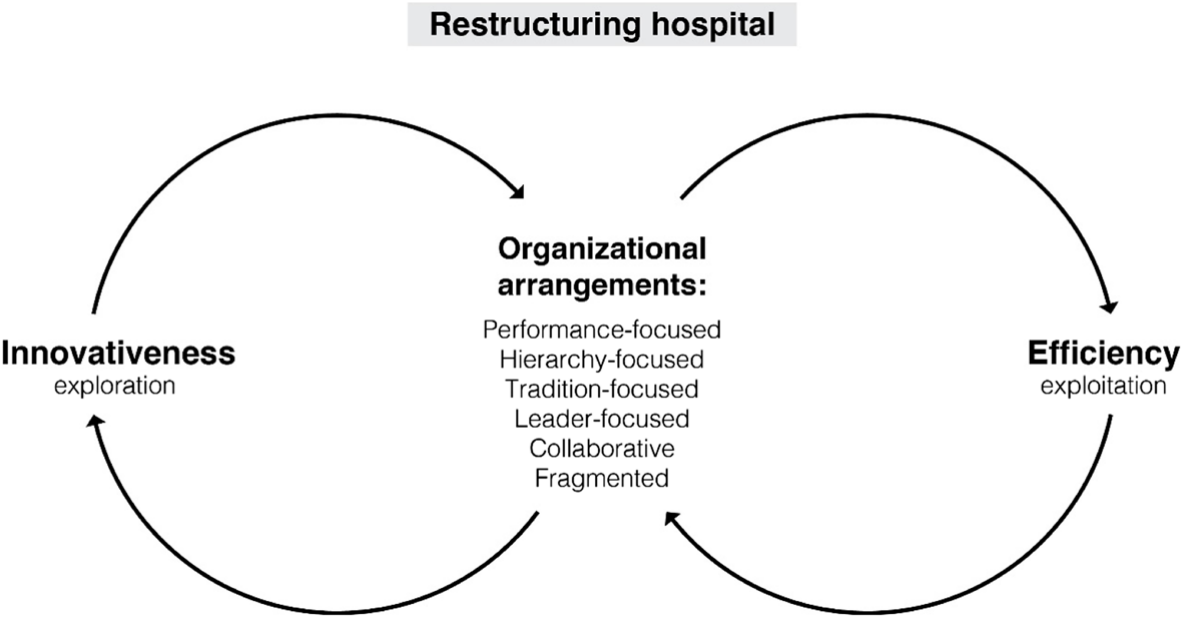
Illustrative summary of the hypothesized relationships

Figure 1 provides a summarizing conceptual model of the relationships discussed above. Since limited empirical evidence exists regarding the organizational arrangements as just described, we next turn to testing the hypotheses in a hospital that is undergoing a notable restructure.

### Research context, sample, and data

There had not been a prior examination, so we took an exploratory stance in our examination, treating our setting as a quantitative case study [73]. We conducted our study in a single organization, examining the perceptions of individuals within that organization through a survey.

We selected a hospital in Finland as our research site to explore the relationships between organizational arrangements and efficiency and innovativeness in the context of restructuring organizations. This allowed us to gain a view of the issues of interest in a pluralistic, public sector health care organization. The single site survey enabled us to examine employee perceptions and attitudes to management across a group of employees (rather than assume that the organization as whole or its individual divisions would consistently reflect some specific organizational form) and thereby observe the possible co-existence of multiple organizational arrangements and their connections to perceptions about innovativeness and effectiveness at an individual level.

In this hospital, a comprehensive organizational restructuring endeavor was launched about a year before our data collection. The restructuring had explicit aims to increasing effectiveness by 10–15% and to improve the quality of care, the speed of the availability of services, and patient safety. The restructuring would affect about 4500 staff members and 730 000 citizens. The hospital is located in a country with an ageing population, increasing service demands, and timely societal demand to develop public sector health care services and their availability, timing, quality, and effectiveness. In this context, the hospital restructuring included aspects ranging from process enhancement and digitalization of care to renovations and the complete rebuilding of outdated buildings, and – as a relevant aspect of our study – to reorganizing the work processes. Notably, there was an explicit aim to increase collaboration and cooperative modes of operation.

For our study, the first author developed the *Workplace Relations Survey* in collaboration with Professor Paul S. Adler. The questions in the survey build on the sociological theories of Weber [11] and his typology of social action. The measurement scales were originally developed by Paul S. Adler and Charles Heckscher [24] to evaluate working relations at primary care practice sites, and they later further developed these to study hospitals in the US. Thereafter, the instrument was further modified and contextualized to measure working relations, and to match our framing of organizational arrangements assuming the importance of individuals’ perceptions [37] in the context of the Finnish health care system. In this process, the point of view was changed from a unit-specific situation of the organizing to perceptions of the individual hospital employees on their organization. This was done because individual-level perceptions may have even more power than the actual situation [3, 22], because the perceptions may not follow the boundaries of units, and since public and private hospitals have inherently different ways of organizing their functioning and services [74]. To ensure accuracy of statements in the survey, in Finland, two senior physicians and two members of the hospital’s development project team conducted the contextualization and modification of the survey together with the first author.

The questions in the survey covered individual respondents’ perceptions of clinical and administrative processes; relationships between administrative and clinical personnel and between doctors, other staff, and patients and management; resources; the recruitment of newcomers; and rewards and values. The design of the questionnaire allowed for individuals having different impressions of the extent to which the organization had specific traits, irrespective of whether they came from the same or other units. We believe that these subjective evaluations, when gathered widely, provide a holistic picture of the organization than objective measures collected from fewer, specific informants or points.

The survey was administered by using an electronic survey platform. Approval to conduct this research was granted by the hospital’s senior management in line with the ethical rules for undertaking research in hospitals [75]. All procedures performed in this study were in accordance with the ethical standards of the regional research committee and with the 1964 Helsinki Declaration and its later amendments or comparable ethical standards. The project manager of the restructuring process in the hospital sent the link to the survey to the managers of all major operative units of the hospital. These managers forwarded the survey to their subordinates with a cover letter indicating that responding to the survey was voluntary but important for collecting information for the ongoing restructuring efforts of the hospital. Participant consent was implied by submitting a response to the survey. Eventually, our data comprised 447 responses from the hospital. The respondents represented 19 professional groups across the hospital ranging from doctors and nurses to physiotherapists and operational management.

### Methods of data analysis and measures

The hypothesis testing was carried out by means of hierarchical regression analyses (conducted with SPSS 27), using an examination of the descriptive statistics and correlation analyses to provide the initial insight. This approach was adopted due to the exploratory nature of our study, and the nature of the measures. The approach chosen for the empirical examination was guided by the attempt to capture perceptions of individuals (rather than objective information), and to gain an initial understanding of the nature of the relationships between the individual constructs of interest, rather than causalities or more general patterns (which we consider a relevant topic for future research).

In line with established practices, we strived to make our measures convergent, discriminant and nomological. For development of the measures, and to secure the construct validity and reliability, we utilized factor and reliability analyses suitable for reflective measures. For organizational arrangements, the measures followed earlier theorization (see especially [12, 23]) and, accordingly, formative measures were used. Guidelines described in Diamantopoulos and Winkelhofer [76] were followed.

#### Dependent variables

*Innovativeness* as perceived by individuals was measured as a construct comprising six items (e.g., “Our organization…” “… frequently implements entirely new processes”; “…frequently develops products and services that are completely new to our organization”; “Developing new technologies [is considered important]”). The Cronbach alpha value for this construct is 0.804. The Composite reliability (CR = 0.866) and Average variance extracted (AVE = 0.520) meet the threshold requirements of 0.70 and 0.50 suggested in Fornell and Larcker [77]. In addition, comparing the square root of the AVE with the correlations of latent constructs, each construct’s AVE has a greater value than the correlations. Respectively, the subjectively evaluated *Efficiency* measure comprised six items that covered, e.g., “Reducing costs” and “Ensuring quality conformance” as important issues, and continuous improvement of processes taking place in the organization (alpha = 0.637; CR = 0.856; AVE = 0.50). While the reliability is not as strong for perceived efficiency as it is for innovativeness, we still meet the 0.60 threshold value for newly developed, original constructs [78, 79].

#### Independent variables

The independent variables were constructed from items that showed respondents’ perceptions on various elements identified as relevant constituents of working relations in earlier research [12], for the theoretical logic behind the specification. These elements were (1) administrative work procedures, (2) clinical work procedures, (3) relationships with other units, (4) new recruits, (5) authority relations, (6) rewards, (7) values, (8) relationships between doctors and other staff, and (9) relationships between staff and patients. More specifically, the respondents were asked to evaluate these nine elements against indicators that captured the extent of each organizational arrangement showing in the elements in question (1–9 above). For instance, the respondents assessed the presence of *hierarchy-focus* in values by indicating how accurate the following statement was: “people think that what we should care most about is respecting the rules and procedures.” More widely, hierarchy-focused form comprises (dis)agreement of the respondents on approvals being required for changes in administrative and clinical processes; on relationships being handled through formal policies and procedures; on recruitment favoring structure-oriented people; on authority focusing on monitoring of rule-following; and on rewards being based on formal duties. Correspondingly, *performance-focused* organizational arrangement, for example, comprises (dis)agreement regarding individuals having autonomy in achieving administrative and clinical goals and targets; relationships being handled as if business transactions; recruitment favoring result-oriented people; authority focusing on setting of goals; rewards being based on performance; and values reflecting appreciation of performance. Instructed by theory and statistical analyses, aggregate measures were formed for *performance-focused, hierarchy-focused, tradition-focused, leader-focused, collaborative,* and *fragmented organizational arrangements*. The same elements were accepted for all measures to keep same content in all of the independent variables [76].

#### Control variables

To address potential effects of other factors that have been considered in earlier research to relate to efficiency and innovativeness [80], we also controlled for factors such as *age*, *gender*, *experience in working in the unit* (years within the same unit), and *resources* (*perceived adequacy of resources* measure comprised of 7 items, with Alpha = 0.74). The *work unit* of the respondents was also included as dummies (even if not reported in detail; this information is available from the authors upon request).

## Results

We started our analysis by looking into the descriptive statistics and correlation matrix (see Table 1). In the data, 57% of the respondents were aged between 41 and 60 years, and 75% were female, representing a relatively typical setting in other similar organizations. The correlation matrix indicates that the organizational arrangement types are related to each other to some extent, and that they also connect to innovativeness and efficiency in specific, varied ways. While most connections are positive, fragmented organizational arrangement seems to have negative relationships with resources and efficiency, for example. In fact, outside our core research framework, the perception of the adequacy of resources seems to have a positive relationship with all other organizational forms except for the performance-focused form (for which no relationship could be detected) and fragmentation (showing negative correlation).

**Table 1:** Correlation matrix and descriptive statistics – Initial look into the relationships of organizational arrangements, efficiency and innovativeness

Variable		Mean (S.D.)	2	3	4	5	6	7	8	9	10
1	Experience (years)	11.42 (8.79)	.013	-.057	-.076	-.071	.011	-.026	-.074	.077	-.028
2	Resources	3.07 (.62)		-.035	.107*	.111*	.217**	.502**	-.559*	-.023	.078
3	Performance-focused	3.03 (.55)			.466**	.344**	.253**	.034	.147**	.304**	.266**
4	Hierarchy-focused	3.43 (.51)				.446**	.237**	.137**	.000	.190**	.216**
5	Tradition-focused	3.50 (.44)					.315**	.001	.013	.044	.089
6	Leader-focused	3.23 (.55)						.571**	-.229**	.195**	.283**
7	Collaborative	3.30 (.68)							-.638**	.136**	.337**
8	Fragmented	2.58 (.65)								-.057	-.232**
9. Innovativeness		3.15 (.79)									.591**
10. Efficiency		3.67 (.61)									

^*^p < .05; **p < .01;

Apart from Experience, the constructs in the Table are measured with Likert scales from 1–5 reflecting individuals’ perceptions and with higher values indicating more of the feature in question

Following the examination of the correlation matrix, hierarchical regression analyses were used to explore the relationships between the constructs more closely. Prior to this, statistical criteria were checked to ensure that there had been no violation of the underlying assumptions of regression analyses. Most values of the variable inflation factor (VIF) were below 3 (with the highest value being 2.7) and all of them were below the threshold of 10 [81]. This suggests that there was no immediate multicollinearity issue [76]. The scatter plots of the residuals, histograms, and normal probability plots were also checked, and they showed normal distributions. The Breusch-Pagan test was also run, showing that heteroscedasticity was not present and that the residuals were normally distributed. Therefore, there was no immediate need to be concerned about heteroscedasticity and non-normality.

The regression analyses were carried out by entering the control variables first, and then adding the main constructs. We chose to enter all organizational arrangement types simultaneously since according to the theory, they are not mutually exclusive but can be present simultaneously to different extents. Table 2 shows the findings from the regression analysis.

**Table 2:** Regression analyses – relationships between organizational arrangements and innovativeness and efficiency

*Variables*	*Innovativeness*		*Efficiency*	
	**Model 1**	**Model 2**	**Model 3**	**Model 4**
1. Age	.005(.040)	-.020(.038)	-.039(.031)	-.057(.028)*
2. Gender	.287(.099)**	.185(.095)^a^	.160(.077)*	.069(.071)
3. Experience	.006(.005)	.009(.005)^a^	.001(.004)	.002(.004)
4. Resources	-.014(.061)	-.131(.071)^a^	.094(.047)*	-.135(.053)*
5. Performance-focused		.411(.075)**		.275(.056)**
6. Hierarchy-focused		.135(.083)^b^		.119(.061)*
7. Tradition-focused		-.201(.094)*		-.111(.070)
8. Leader-focused		.103(.084)		.064(.062)
9. Collaborative		.120(.081)		.232(.061)**
10. Fragmented		-.080(.077)		-.162(.057)**
11. Unit	incl	incl	incl	incl
F	2.810*	7.715**	2.295^a^	12.207**
R^2^	.025	.153	.021	.222
R^2^ Adj	.016	.133	.012	.204

Std. errors in parenthesis;

^*^p < .05; **p < .01, ^a^*p* < .10; (^b^*p* = .103)

The regression analysis suggests that Hypotheses 1a and 1b on the positive relationships between performance-focused organizational arrangement and innovativeness and efficiency are supported. Hypotheses 4a and 4b expecting Leader-focused organizational arrangements to be connected to efficiency and innovativeness are not supported.

**Fig. 2:**
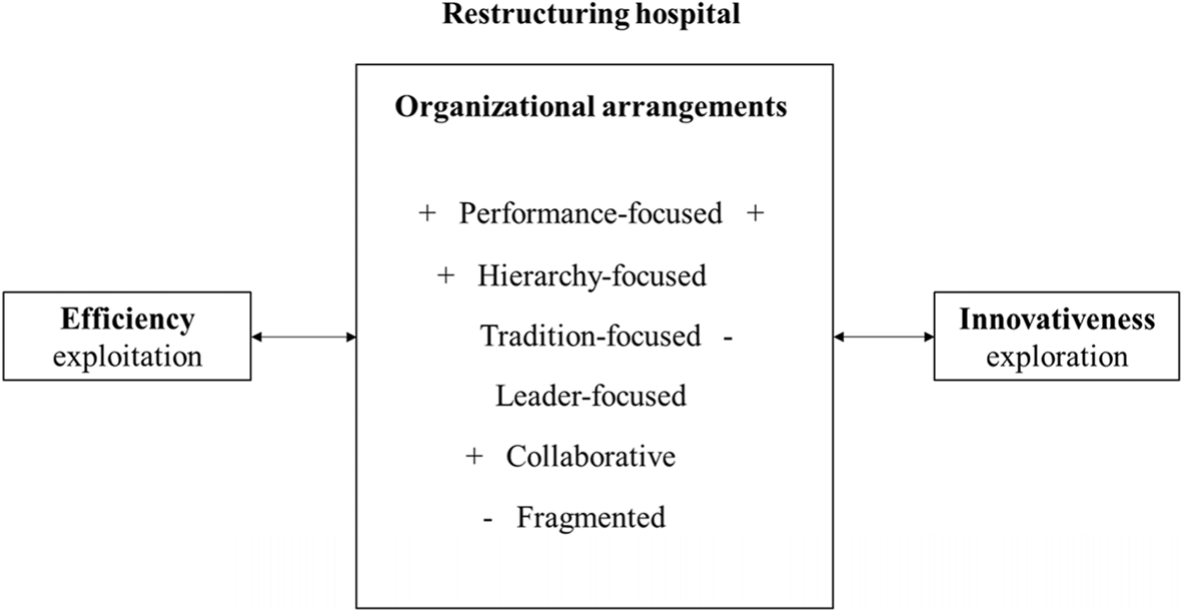
Relationships between organizational arrangements, and innovation and efficiency

For other organizational arrangements, the findings match expectations with regard to one of the perceptions of innovativeness and efficiency, but not the other. First, hierarchy-focused organizational arrangement (H2a and H2b) seems to be positively related to efficiency, but not to innovativeness (the significance of the test showing a positive relationship rather than the expected negative relationship is marginal). Second, the tradition-focused organizational arrangement is negatively related only to innovativeness (H3a). Efficiency (H3b) does not reach statistical significance with regard to levels of traditionalistic approaches being present in the study organization. Collaborative organizational arrangement is positively related only to efficiency (H5b); the relationship with innovativeness (H5a) is not statistically significant. Finally, the fragmented type is negatively related to innovativeness (H6a), but there is no significant relationship between high levels of fragmentation and efficiency as perceived by individuals (H6b). Figure 2 summarizes the findings.

## Discussion

Despite the proliferation of research on change and restructuring in fields such as health care [13–15], and on efficiency and innovativeness in more particular [82], there has been a paucity of effort to produce knowledge on how different organizational arrangements capturing subjective factors connect to reaching innovativeness and efficiency at the individual level in organizations undergoing restructuring. In particular, there is little empirical evidence of the individual-level perceptions that become pivotal when aiming at restructuring that promotes both innovativeness and efficiency. To narrow this gap, as well as to understand the potential of health care restructuring efforts for supporting high levels of performance and organizational change, we have explored relationships between organizational arrangements and innovativeness and efficiency in a public sector hospital. Our empirical findings show that various organizational arrangements are present in the restructuring hospital, and that these have their specific relationships with innovativeness and efficiency [12, 16].

First, our findings reveal that the *performance-focused* organizational arrangement is highly relevant with regard to efficiency and innovativeness [50, 51]. Our results support Hypotheses 1a and 1b anticipating the emergence of positive relationships. The theoretical discussion points in this direction [12, 82]: as it is considered that the performance-focused organizational arrangement captures the relevance of high performance for employees in meeting organizational goals (including those set by senior management and reflecting entrepreneurial orientation), the finding seems logical in a study conducted in an organization that has set efficiency and innovativeness as its central, explicit goals: these elements become more visible to the individuals.

Second, our study showed that *leader-focused* organizational arrangement does not play a notable role in the hospital organization’s search for innovativeness and efficiency. Considering that the hospital we studied is relatively large, the influence of influential leaders (e.g., doctors) may be limited [12, 38, 83]. Vergauwe and colleagues [84] also noted that highly charismatic leaders might have inadequate skills and therefore be less effective in helping to reach organizational goals. It may be that not finding a significant relationship is a result of opposite organizational forces playing a role; leaders may inspire others to work more efficiently and creatively, but then it also may be that competing interests emerge, especially in a large organization. For example, innovation is often prone to this type of challenge [85].

Third, our empirical findings suggest that *hierarchy-focused* organizational arrangement is positively related to efficiency. If not overly limiting, hierarchy, structures, and bureaucracy can provide individuals with the direction for improving the practices and processes [12, 54]. Somewhat unexpectedly, hierarchy-focused form was not found to be harmful to innovativeness, but rather, the sign was actually positive (even if not statistically significant). Innovation too, calls for structures and hence, hierarchies and bureaucracy are not self-evidently innovation-hampering [10, 52, 86]. In line with this, Jønsson and colleagues [39] suggest that leaders should not merely provide autonomy and leave subsequent steps and decisions to employees [34]. In fact, as innovativeness had already been accepted as the explicit goal in our study context, it is likely that it had been incorporated in the hierarchical elements also. However, this aspect invites further examination.

Fourth, in line with our expectations, the analysis highlighted that the *tradition-focused* organizational arrangement is negatively related to innovativeness. However, while the sign is negative for efficiency, our analysis showed no statistical significance. In hospitals, efficiency may already have been built into the established practices and processes at the level of values [87], in which case a negative relationship will not necessarily emerge. This issue might be worth further examination in other hospitals and health care organizations.

Fifth, somewhat surprisingly, our analysis suggested that *collaborative* organizational arrangements are positively related only to efficiency, while the relationship with innovativeness is not statistically significant. Although it could be expected that sharing knowledge and cooperation yields genuinely new combinations of knowledge [36], our case underscores something in the hospital that limits this. A question arises if the threshold level of overlap and shared meaning required for innovation to emerge in collaborative work [68] are missing in an organization with historical professional boundaries [4, 62]. Also, the separate resource allocation to distinct result units and the competition between these have been identified as factors creating obstacles and boundaries that inhibit such that would support realizing innovation and learning [43]. In the light of discussions with the staff at the hospital studied in this study, this is a plausible explanation: collaboration is more tuned towards cost savings and efficiency. Yet another explanation for not finding a significant relationship potentially is that our measure highlighting consensus and other such features as characteristics of a collaborative organizational arrangement also captures innovation limiting factors: too much like-mindedness can also limit the emergence of novel ideas [61]. The positive relationship between collaborative organizational arrangement and efficiency could be explained by the fact that collaborative work has been targeted at solving acute problems. This is manifested in practice as joining forces in alarming crises or in mundane situations, such as when a team needs to cover for their colleagues when they take sick leave.

Finally, *fragmentation* can be considered to be a negative phenomenon [65, 67] and in our study, individual perceptions of fragmentation are negatively related to perceived efficiency. On the other hand, the negative connection does not apply to the same extent to innovativeness. We suspect that this is due to countering forces. Fragmentation could also be beneficial when considering innovativeness in the sense that dissimilar views and a suitable amount of disagreement can bring out new ideas [22, 71]. It may also be that fragmentation has become an accepted feature in the pluralistic organization and is no longer problematized. However, this issue calls for deeper examination.

In addition to these insights into the various organizational arrangements, our findings indicate that as they are subjective, multiple organizational arrangements co-exist in the hospital we studied, meaning that there are varied views of the organization and working relations within [22]. Whereas some organizational members may consider the organization to be performance-focused, others (even in the same unit) may focus on hierarchies or leader(s)’s role (potentially as the driver of performance). The ongoing restructuring plays a role here. Prior studies have established that the new modes of organizing cannot easily or quickly replace the historically established forms. Collaborative organizational arrangement seems to be the direction pursued, but it clearly differs from the other, prevailing organizational arrangements. Our empirical findings suggest that although collaboration and being in networks are gaining momentum as solutions to innovativeness and efficiency [43, 67], reaching collaborative organizational arrangements takes time [88], and other forms of organizational arrangements can support the efficiency and innovation related goals during the change process if they are allowed to coexist. These findings connect with wider theoretical discussions on the historical embeddedness of organizations and their change processes [89] and on innovation management [29, 62].

In addition to the novel insights, the limitations of our study provide opportunities for future research. An obvious limitation is that we only studied the relationships of interest within one organization with its own specific features and situation. This limits the generalizability of our findings. While the approach chosen serves the purposes of this study well (especially the attempt to study the topic of interest in the individual level), further research is needed that tests these aspects in other settings (such as private hospitals and varying healthcare organizations in other countries). Additionally, while we added some controls in this study, there are opportunities to consider the role of other factors, and a need to do so. These include the organizational units’ role and comparisons between these, and the professional background of the respondents, for instance. Likewise, the role of resources (taken as a control factor here) might be more complex, and likely deserves more attention. As the first attempt to study the organizational arrangements’ relationship with efficiency and innovativeness, focusing especially on the individual level perceptions, the empirical setting was deliberately kept relatively simple. Hence, while our study provides the initial insight on the existing relationships and hopefully stimulates further questions, future studies are needed to examine causalities and more sophisticated models.

There might be a need to study other moderating or mediating relationships. Using different measures is also possible; objective measures could be used to assess efficiency and innovativeness. However, the setting would then be notably different from ours which had its focus on perceptions, and in a hospital context that differs from a corporate one, the choice of measures should be carefully considered. Finally, as a cross-sectional study always has its limitations in terms of determining causal relationships, we encourage exploration of reverse causalities and suggest longitudinal studies that can provide valuable information on the development trajectories and patterns that emerge throughout restructuring processes. Studies of this kind can be conducted in other healthcare organizations and may involve mixed methods to gain both in-depth insight and a better understanding of the nature of relevant relationships.

## Conclusions

### Theoretical contributions

Our findings contribute to earlier discussions on the ways in which efficiency and innovativeness can be achieved in connection to radical changes and restructuring efforts, especially in the context of professional, pluralistic organizations [7, 17, 18] Specifically, our study extends earlier theorizations by addressing the connections between organizational arrangements as perceptions of working relations [37, 64] and innovativeness and efficiency at the individual level [34], calling for more this kind of research, and by providing empirical evidence of these aspects in the context of health care organizations.

Our findings suggest that being subjective, multiple organizational arrangements co-exist in pluralistic organizations. They further indicate that when the specific aim of the restructuring is not only to achieve costs savings, but simultaneously to promote efficiency and innovativeness, this co-existence is likely to enable taking the necessary steps to reach the outcomes pursued [3, 12, 41, 90]. Hence, our study advances what is known about how hospital management and medical professionals collectively arrange their practices when facing multiple demands for change and for undergoing a restructuring process.

Our findings show that while cooperation and collaboration are pursued for their obvious benefits [6, 14, 16, 49], collaboration is not necessarily universally optimal, and that there are varying directions that could be taken that are viable [61, 71]. In fact, if collaboration and convergence are pursued too aggressively, without acknowledging existing ways of organizing, it may be that resistance increases and that the organization’s members return visibly and strongly to the traditions. It may be that fragmentation will increase and disrupt the change processes. Together, these developments jeopardize both efficiency and innovativeness.

To sum up, our study can be considered to be a step in the theory development in the sense that it points toward benefits of varied organizational arrangements, shows the limits of collaboration, and illustrates the challenges fragmentation and stagnation can pose.

### Practical implications

Our study has specific implications for hospital and health care management practices and policy making. Our findings indicate that innovativeness and efficiency can be promoted and supported with varying ways of management [41]. In particular, adequate structures and direction, combined with a search for competitive solutions and suitable level of rivalry could be considered to be useful management strategies in hospitals where professional and expertise-based boundaries may be relatively difficult to permeate, and where establishing collaboration is not straightforward. The findings indicate that restructuring and change benefit from allowing multiple perceptions to be accommodated in an organization instead of pushing for any one organizational arrangement [3, 90]. In fact, while many studies have recently praised collaborative innovation, based on our findings, it is important to have a culture that appreciates good performance, allows individuals to maintain their own views to ensure person-organization fit, and ensures adequate structures to support fitting levels of collaboration at each time during the change processes.

Policy making benefits from acknowledging these issues, especially in producing guidelines and norms, as well as training and education for health care providers that do not push them to the state of fragmentation. In our case, the hospital represents public sector actors, thereby showing direction specifically for these types of actors. Restructuring an organization – especially one that has historical background as a professional organization – does not need to reach fully collaborative forms to be able to produce social innovation or function efficiently in the public sector health care [91]. Our findings illustrating how organizational arrangements connect to reaching innovativeness and efficiency can support balanced restructuring of health care sector more widely, and ease identifying the ways to deal with the turbulent and contested policy context.

While further examination is still needed, our study brings forth the idea that the (remnants of) historical ways of organizing and social action continue to play a role in current hospitals, and that they may surface in transformation and restructuring situations. How these relate to the goals with regard to innovativeness and efficiency may come as a surprise unless managers and policy makers are prepared to accept that transformation may not be as fast or as complete as expected. This is a point worth carrying forward, and we hope to see our study as one relevant step on that path.

## Data Availability

The datasets generated and/or analysed during the current study are not publicly available due to confidentiality having been guaranteed to the respondents and an agreement with the creators of the original survey instrument about its disclosure. However, data are available from the authors upon reasonable request, conditioned by the permission of the hospital and the creators of the survey instrument.
